# Interaural-time-difference thresholds for broad band-limited pulses are affected by relative bandwidth not temporal envelope sharpness

**DOI:** 10.1121/10.0008971

**Published:** 2021-12-09

**Authors:** Paul G. Mayo, Philip C. Saunders, Matthew J. Goupell

**Affiliations:** Department of Hearing and Speech Sciences, University of Maryland, College Park, Maryland 20742, USA paulmayo@umd.edu, psaund99@umd.edu, goupell@umd.edu

## Abstract

Humans are sensitive to interaural time differences (ITDs) conveyed by slow modulations on high-frequency carrier signals. Sensitivity appears to be affected by temporal envelope sharpness, but it is unclear if there is a limit to which sharpness affects sensitivity. Pulse trains were varied in relative bandwidth (re: critical bandwidths) and center frequency. ITD sensitivity increased with increasing bandwidth. There was no effect of center frequency when relative bandwidths were analyzed, suggesting that the temporal envelope sharpness (concomitantly absolute bandwidth in Hz) did not affect performance. Rather, sensitivity was most easily explained by recruitment of additional auditory channels.

## 1. Introduction

Interaural time differences (ITDs) are an important cue for sound localization in the horizontal plane (Macpherson and Middlebrooks, 2002). Humans are exquisitely sensitive to ITDs in the low-frequency (<1500 Hz) temporal fine structure, as well as for high frequencies (>1500 Hz) when there are slow (<300 Hz) envelope modulations (Hartmann, 2021). Envelope ITD sensitivity has been measured using a variety of modulators, including sinusoidal-amplitude-modulation, raised-sine envelopes, and Gaussian envelopes (Stecker *et al.*, 2021). ITD sensitivity for such signals is affected by the modulation rate, modulation depth, and temporal envelope sharpness (Bernstein and Trahiotis, 2009, 2010; Klein-Hennig *et al.*, 2011; Laback *et al.*, 2011).

Band-limited acoustic pulses have been used as a simulation of the electrical pulse trains presented to cochlear-implant users, where a primary goal is to approximately match the wide bandwidths that occur with electrical stimulation and current spread (Goupell *et al.*, 2009). An advantage of using such stimuli is that it is possible to parametrically investigate factors underlying ITD sensitivity without the variability inherent to the clinical cochlear-implant population (Kan and Litovsky, 2015). One assumption, however, is that the temporal-envelope onset of these stimuli is sufficiently sharp to approximate the instantaneous onset of an electrical pulse. The purpose of this study was to return to that assumption, as it must be met for these single acoustic pulses to be a valid simulation of electrical stimulation. Specifically, is the temporal envelope of band-limited acoustic pulses sharp enough that it does not appreciably impact ITD sensitivity and produce confounds for interpreting data?

Any change to the spectral properties of a narrowband acoustic signal will have concomitant physical changes to the temporal properties and vice versa. Larger bandwidths consequently produce sharper temporal pulse envelopes, and have been utilized in multiple studies where the bandwidth was kept approximately constant in millimeters to simulate current spread of a cochlear-implant (CI; Majdak and Laback, 2009), which corresponds to an approximately constant number of channels or equivalent-rectangular-bandwidth (ERB) rate (Moore and Glasberg, 1983). In other words, as the center frequency increased, the individual pulses became sharper (as ERBs are wider absolute bandwidths at higher center frequencies), which has the potential to improve ITD sensitivity. Likewise, Goupell *et al.* (2013) used stimuli that had mismatched center frequencies but kept the bandwidth constant in millimeters, meaning that the stimuli across the ears had different absolute bandwidths and thus, different amounts of temporal envelope sharpness.

Therefore, the goal of this study was to determine if ITD sensitivity could be better explained by temporal envelope sharpness or relative changes in the frequency domain, as this would help better understand the stimulus parameters that affect simulations of pulsatile electrical stimulation. To do this, we employed very low-rate [10 pulses per second (pps)] acoustic pulses to avoid temporal overlap and any rate effects, and then varied the bandwidth (in ERB) of these acoustic pulses for different center frequencies with different channel or critical bandwidths. We hypothesized that if temporal envelope sharpness was the driving factor behind ITD sensitivity, there would be an interaction between the physical sharpness/bandwidth scale in Hertz and center frequency. This interaction would be due to the saturation in sharpness/bandwidth that occurs from peripheral filtering for stimuli with bandwidths >1 ERB. There was also an alternative hypothesis that posited continual improvement with increasing relative bandwidth from recruitment of additional auditory channels (i.e., across-frequency combination of information).

## 2. Methods

### 2.1 Listeners

There were nine young normal-hearing adult listeners (23–38 y, average = 23.7 y) with pure-tone air-conduction thresholds ≤20 dB (re: hearing level) for octave frequencies from 0.25–8 kHz. Because some of the stimuli were presented at very high frequencies, we also tested hearing thresholds at 12 kHz. The listeners had no asymmetry >10 dB at any tested frequency.

### 2.2 Stimuli

**Fig. 1.**
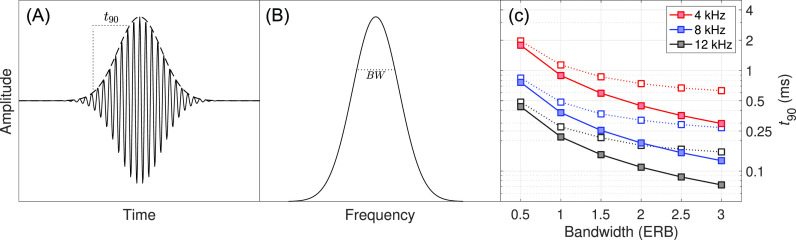
Panel (A) shows a single Gabor pulse with the Gaussian-shaped temporal envelope and $t_{90}$ metric highlighted. Panel (B) shows the spectral representation of a single Gabor pulse with the Gaussian-shaped spectral envelope and reciprocal measure to the $t_{90}$ metric, absolute bandwidth in Hz, highlighted. Panel (C) plots the $t_{90}$ of the stimuli used in this experiment. Closed symbols represent $t_{90}$ of the broadband stimuli. Open symbols represent $t_{90}$ of the stimuli after being processed by a Gammatone peripheral filter at the carrier frequency of the pulse train.

The stimuli were trains of Gaussian-envelope–modulated cosine pulses, or “Gabor” pulses (Gabor, 1947; e.g., Fig. 1). The sinusoidal carrier had a frequency of 4, 8, or 12 kHz. The bandwidth of the pulses was manipulated so that the −3-dB bandwidth was 0.5, 1, 1.5, 2, 2.5, or 3 ERBs (according to Moore and Glasberg, 1983) for their respective center frequencies, making 18 total conditions. All trains were generated in the frequency domain using custom software written in matlab (the Mathworks, Natick, MA). The trains had a pulse rate of 10 pps, low enough to avoid any substantial pulse overlap, reduction in modulation depth, or interaction between center frequency and ITD sensitivity (Goupell *et al.*, 2019). Each pulse train had a duration of 300 ms and thus, consisted of three pulses. Stimuli were created at a 400-kHz sampling rate, allowing for a 2.5-*μ*s ITD resolution, and down-sampled to 100 kHz after the ITD was applied. The left and right earphones were independently calibrated so that a long-duration, 10-pps, 8-kHz, 1-ERB stimuli measured 65 dBA; the other pulse trains were spectral peak normalized because of the desire to better understand the stimuli from Goupell *et al.* (2013).

Binaurally uncorrelated masking noise was presented to mask possible low-frequency distortion products. The masker was Gaussian white noise with a 1st-order Butterworth low-pass filter at 0.2 kHz, and a 3rd-order Butterworth low-pass filter at 1 kHz. The masking noise was presented at 40 dBA.

The sharpness (or attack time) of a single Gabor pulse was quantified using the *t_90_* measure, the time difference between 10% and 90% of the peak temporal envelope value (Giannoulis *et al.*, 2012). The *t_90_* (in seconds) of a Gabor pulse can be precisely calculated as a function of its −3-dB bandwidth (in Hz):

$$t_{90}=\sqrt{-2*{\left(\frac{\sqrt{2*\ln\left(\sqrt{2}\right)}}{\mathrm{BW}*\pi }\right)}^{2}*\;\ln\left(10/100\right)\;}-\sqrt{-2*{\left(\frac{\sqrt{2*\ln\left(\sqrt{2}\right)}}{\mathrm{BW}*\pi }\right)}^{2}*\;\ln\left(90/100\right)\;}\approx \frac{0.447}{BW}.$$ (1)

This equation can be modified to calculate the time difference between arbitrary points on the temporal envelope by substituting 10% and 90% for alternate starting and ending proportions, respectively.

### 2.3 Procedure

Stimuli were passed through a digital-to-analog converter (RP2.1, HB7, Tucker Davis Technologies, Alachua, FL). They were presented over circumaural headphones (Sennheiser HD650, Old Lyme, CT) to listeners seated in a double-walled, sound attenuating booth (IAC, North Aurora, IL).

Listeners performed an adaptive ITD left–right lateralization discrimination task using a custom matlab interface. The listeners were presented a 0-*μ*s ITD stimulus, 300 ms of silence, then, the same stimulus with a randomly left or right leading ITD. Listeners were asked if the second sound (with the non-zero ITD applied) was perceived as left or right of the first. No correct answer feedback was given.

A 3-down-1-up adaptive procedure was used to find ITD thresholds (Levitt, 1971), with an initial ITD of 2500 *μ*s and step factor of 2. The step factor decreased to $\sqrt{2}$ after the second reversal. Staircases ended after 10 reversals, and thresholds were calculated as the geometric mean of the last six reversals. Each of the 18 conditions was measured three times in uniformly randomized blocks, resulting in 54 adaptive staircases per listener. The final reported threshold was the geometric mean over the three individual track measurements.

All listeners completed a training phase prior to the main experiment. Training consisted of at least three staircases with correct answer feedback using the 8-kHz center frequency and 1-ERB bandwidth stimuli. After the third staircase, a running average of all previous thresholds were compared to the most recent threshold. Training stopped when the most recent threshold was within ±15% of the moving average, indicating performance saturation. All listeners completed between three and eight staircases (mean = 4.8) before they moved on to the main experiment.

## 3. Results

**Fig. 2.**
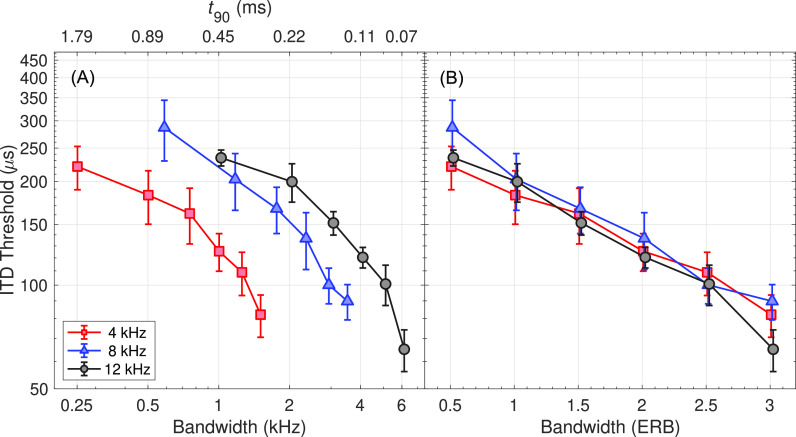
Panel (A) shows the data as a function of absolute bandwidth in Hz (and therefore, also as a function of $t_{90}$) for the three different center frequencies. Panel (B) shows the data as a function of relative bandwidth in ERB.

The results of the experiment are shown in Fig. 2, where panel (A) is plotted as a function of absolute bandwidth in Hz (and therefore, also as a function of $t_{90}$) and panel (B) is plotted as a function of relative bandwidth in ERB. If increasing pulse sharpness influences the ITD thresholds for these already sharp stimuli, then the curves for the three different center frequencies should be aligned in Fig. 2(A), as absolute bandwidth/sharpness is a property independent of center frequency for stimuli with such a low rate, and then diverge when sharpness plateaus from peripheral filtering [see the open points in Fig. 1(C)]. No characteristics of this trend were observed. If the relative bandwidth and number of auditory channels influences the ITD thresholds, then the curves for the three different center frequencies should be aligned in Fig. 2(B), which appeared to be the case. Therefore, a two-way repeated-measures analysis of variance with factors bandwidth (in ERB) and center frequency was conducted. A Greenhouse–Geisser correction was used when the assumption of sphericity was violated. The ITD thresholds improved with increasing bandwidth [F(1.5, 13.0) = 30.4, *p < *0.0001, $\eta_{p}^{2}$ = 0.79]. *Post hoc* paired two-sample t-tests that were Bonferroni-corrected for 15 comparisons revealed that all bandwidths were significantly different from each other (*p < *0.05), except that the 1- and 1.5-ERB bandwidth were not different (*p = *0.10) and 2- and 2.5-ERB bandwidth stimuli were not different (*p = *0.068). The effect of center frequency was not significant [F(2, 16) = 0.68, *p = 0.52*, $\eta_{p\;}^{2}$= 0.08] and the bandwidth × center frequency interaction was not significant [F(2.6, 21.0) = 0.92, *p = *0.44, $\eta_{p\;}^{2}$= 0.10].

Finally, the data in Fig. 2(B) were fit to find the rate of change in ERB. We found a slope of −0.20 (95% confidence interval = [−0.22, −0.17], $R^{2}=0.95$) for log(ITD) versus ERB and a slope of −0.61 (95% confidence interval = [−0.73, −0.50], $R^{2}=0.89$) for log(ITD) versus log(ERB).

## 4. Discussion

The purpose of this study was to determine if increased pulse sharpness improved ITD sensitivity for relatively sharp stimuli, often used in CI simulations (e.g., Majdak and Laback, 2009; Goupell *et al.*, 2013), or if the increased sensitivity could be better explained by changes in the frequency domain. We found that ITD sensitivity improved with increasing bandwidth in ERB, but there was no effect of center frequency or absolute bandwidth/sharpness. Sensitivity improved even for stimuli with bandwidths >1 ERB, where sharpness plateaus due to peripheral filtering [see Fig. 1(C)]. This supports the alternative hypothesis that relative bandwidth and auditory channel recruitment, rather than pulse sharpness, affected performance. Previous studies have investigated the effect of envelope sharpness on ITD sensitivity using exponential-shaped modulation (Laback *et al.*, 2011) and raised-sine envelope manipulations (Bernstein and Trahiotis, 2012). However, the stimuli employed here have envelopes far sharper than those of previous studies. It is possible that sharpness has a direct but bounded effect on ITD sensitivity, recruiting additional channels increases sensitivity, and the stimuli used here exceed the sharpness limit. Regardless, this interpretation supports the notion that pulse trains with relatively large bandwidths are reasonable cochlear-implant stimulations from the standpoint that they appear to have envelopes sharp enough to not have a confound compared to the instantaneous pulse onsets that occur with electrical stimulation.

The ITD thresholds measured were relatively high compared to those reported for stimuli with higher rates (Bernstein and Trahiotis, 2009, 2010), and more in line with those studies using only a few pulses (Hafter and Dye, 1983). Increasing the bandwidth of a high-frequency carrier narrowband noise (Bernstein and Trahiotis, 1994) and pulse trains (Goupell *et al.*, 2013) improves ITD sensitivity. Binaural models that predict performance based on the normalized cross-correlation function after physiologically inspired transformations (auditory filtering, compression, rectification, low-pass filtering capturing neural refractoriness) often focus on temporal aspects of the stimuli and choose a single best channel for describing performance, which is often assumed to be at the center frequency; however, sometimes it is required to move to an off-frequency channel at a higher frequency to obtain a sharper stimulus attack for modulated tones (Bernstein and Trahiotis, 2009, 2010) or to a lower frequency to obtain a lower modulation rate for narrowband noises (Bernstein and Trahiotis, 1994). Other models use across-channel integration of correlation patterns to describe intracranial lateralization (Stern *et al.*, 1988; Bernstein and Trahiotis, 2012), and sometimes ITD discrimination (Trahiotis *et al.*, 2001). The current data, which adds the important parameter of center frequency, suggest that changes in ITD sensitivity with bandwidth are predicted by considering multiple channels, consistent with the latter group of studies.

If across-channel recruitment provided an ideal improvement of ITD sensitivity according to signal detection theory, the fitted slope of log(ITD) to ERB would be −0.5 (an improvement of $1/\sqrt{\;n}$, where n is the number of auditory channels recruited) (Hafter and Dye, 1983) compared to the observed slope of −0.2. One explanation for this discrepancy is that the individual Gabor pulses are continuous in the frequency domain. That is, a continuous number of overlapping channels are recruited on either side of the stimulus center frequency when the stimulus bandwidth is extended and therefore, more than one additional channel is recruited per unit change in ERB. Additionally, a requirement for the theoretically ideal slope of −0.5 is that both the abscissa and ordinate be logarithmic units. While ERBs are relatively logarithmically spaced as a function of frequency, a linear change to a single frequency's ERB (e.g., changing from 1–2 ERBs at 4 kHz as done in this study) is a linear change in bandwidth. A steeper slope of −0.61 when fitting against log(ERB) is closer to the theoretically ideal slope of −0.5, but the correct approach and scale are unclear. It could also be the auditory system simply does not perfectly integrate across-channel information, and that recruiting multiple additional auditory channels does not equal a unit increase in information.

There are a few possible confounds to our interpretation. First, these data do not rule out that the improved ITD thresholds were due to off-frequency listening and energy extending into broader auditory channels (which would have sharper impulse responses than the on-frequency channels). However, this does not seem likely as the absolute bandwidth of auditory channels increases as a function of frequency, so the potential increase in sharpness available from off-frequency listening would be greater for higher-frequency stimuli compared to lower-frequency stimuli. This would have resulted in a center frequency × ERB interaction, which was not observed. Likewise, simulations of off-frequency listening reveal that there is no off-frequency filter that would cause the 4-kHz stimuli to reach the sharpness of the 12-kHz stimuli (data not shown). The only way to have equal sharpness across these stimuli is for the auditory system to listen to a lower-frequency channel with the 12-kHz stimuli, one with up to 64 dB less energy than the on-frequency channel and likely subthreshold. Further research investigating ITD sensitivity as a function of the number of auditory channels without the confound of recruiting broader channels is necessary. Second, these data cannot rule out level effects on ITD sensitivity (Dietz *et al.*, 2013). Due to the spectral peak normalization across all bandwidths, it is possible that there was a level-based change in ITD sensitivity as a function of bandwidth. Third, we did not see a plateau in ITD sensitivity for bandwidths ≤1 ERB. If the increased ITD sensitivity was due to the recruitment of additional channels, then ITD sensitivity should remain constant when the stimulus energy is contained within a single channel. However, the spectra of our stimuli were not rectangular, and it is unlikely that energy was contained within a single auditory channel, even for the narrowest bandwidths. Additional research involving multiple stimuli with bandwidths narrower than a critical bandwidth is necessary to verify this theoretical plateau and support our interpretation.
